# Short-lived species move uphill faster under climate change

**DOI:** 10.1007/s00442-021-05094-4

**Published:** 2022-01-06

**Authors:** Joséphine Couet, Emma-Liina Marjakangas, Andrea Santangeli, John Atle Kålås, Åke Lindström, Aleksi Lehikoinen

**Affiliations:** 1 Finnish Museum of Natural History, University of Helsinki, P. O. Box 17, 00014 Helsinki, Finland; 2 Research Centre for Ecological Change, Organismal and Evolutionary Biology Research Programme, University of Helsinki, 00014 Helsinki, Finland; 3 FitzPatrick Institute of African Ornithology, DST-NRF Centre of Excellence, University of Cape Town, Cape Town, South Africa; 4 Norwegian Institute for Nature Research, Torgarden, Postboks 5685, 7485 Trondheim, Norway; 5 Department of Biology, Biodiversity unit, Lund University, Ecology Building, S-223 62 Lund, Sweden

**Keywords:** Avian community, Altitudinal range shift, Life-history trait, Climate change, Scandinavian mountains

## Abstract

Climate change is pushing species ranges and abundances towards the poles and mountain tops. Although many studies have documented local altitudinal shifts, knowledge of general patterns at a large spatial scale, such as a whole mountain range, is scarce. From a conservation perspective, studying altitudinal shifts in wildlife is relevant because mountain regions often represent biodiversity hotspots and are among the most vulnerable ecosystems. Here, we examine whether altitudinal shifts in birds’ abundances have occurred in the Scandinavian mountains over 13 years, and assess whether such shifts are related to species’ traits. Using abundance data, we show a clear pattern of uphill shift in the mean altitude of bird abundance across the Scandinavian mountains, with an average speed of 0.9 m per year. Out of 76 species, 7 shifted significantly their abundance uphill. Altitudinal shift was strongly related to species’ longevity: short-lived species showed more pronounced uphill shifts in abundance than long-lived species. The observed abundance shifts suggest that uphill shifts are not only driven by a small number of individuals at the range boundaries, but the overall bird abundances are on the move. Overall, the results underscore the wide-ranging impact of climate change and the potential vulnerability of species with slow life histories, as they appear less able to timely respond to rapidly changing climatic conditions.

**Supplementary Information:**

The online version contains supplementary material available at 10.1007/s00442-021-05094-4.

## Introduction

During the Anthropocene, ecosystems are experiencing rapid shifts in climate. Recent climate change includes increases in temperature, changes in precipitation patterns and sea levels, decreases in snow cover, and increases in frequency and intensity of extreme events (IPCC 2014). These changes have profound impacts on life on Earth from the level of individuals to species, ecosystems, and biomes (Parmesan 2006; Scheffers et al. 2016; IPBES 2019). At the species level, there are three possible responses to climate change: adaptation, range shift, or/and local or global extinction (Parmesan 2006; Alford et al. 2007; Robinet and Roques 2010).

Under climate change, species can shift their ranges towards higher latitudes and/or altitudes in search for suitable climatic conditions to which they are adapted (Thomas et al. 1999; Walther et al. 2002; Walther 2010; Gillings et al. 2015; Stephens et al. 2016). Patterns of species’ range shifts are consistent with a gradient of decreasing temperatures toward higher latitudes and altitudes (Pautasso 2012). Indeed, various taxa, including insects, mammals, birds and fish, have been observed to shift their ranges to higher latitudes at a rate of 17 km per decade and to higher elevations at a rate of 11 m per decade (Chen et al. 2011).

Species’ ranges, and their altitudinal shift potential, can be determined by the species’ capacity to disperse, establish new populations, and proliferate (Pöyry et al. 2009; Bateman et al. 2013). Such capacities depend, at least partly, on species’ traits (Van der Vaken et al. 2007). The environmental tolerances, such as the climatic conditions and the diversity of habitats that the species are able to exploit, shape species’ ranges (Thompson et al. 1999). Various traits can affect species’ potential to shift their ranges, such that species with higher dispersal capacity, reproductive rate, and degree of ecological generalization should be more able to colonize new suitable habitats (Angert et al. 2011; Laube et al. 2013; Auer and King 2014; Estrada et al. 2016; Lehikoinen et al. 2021). Moreover, migratory species have been reported to have a small range shift potential (Forsyth et al. 2004; Välimäki et al. 2016), probably because they show higher fidelity to breeding and overwintering sites compared to resident species (Bensch 1999). Despite the above-mentioned examples, the effects of species’ traits on range and abundance shift are still unclear. A recent meta-analysis concluded that the “current understanding of species’ traits as predictors of range shifts is limited” (MacLean and Beissinger 2017).

Studying altitudinal shifts of wildlife, both in terms of range and abundance, is particularly relevant and timely from a conservation perspective. Mountains are among the most vulnerable ecosystems on Earth, facing disproportionate impacts of changing climate, while still harboring uniquely specialised, adapted, and range-restricted species (Thompson 2000; Rahbek 1995; La Sorte and Jetz 2010). Compared to other ecosystems, mountaintops typically represent climate refugia that offer only limited space for species to shift in search of optimal conditions, further increasing their extinction risk (Şekercioğlu et al. 2008; Gonzalez et al. 2010; Sirami et al. 2017; Scridel et al. 2018). The few available studies on altitudinal shifts in wildlife report contrasting patterns (Archaux 2004; Popy et al. 2010; Maggini et al. 2011). However, their spatial and taxonomic extents are limited and they are mainly based on presence–absence, rather than abundance data. When quantifying the speed of altitudinal shifts, as well as the relative influence of different traits on altitudinal shifts, the use of abundance data can greatly increase our understanding on the factors that contribute to the vulnerability of mountain species and how they can be conserved (Virkkala and Lehikoinen 2014; Foden and Young 2016).

Here, we use a comprehensive longitudinal dataset of bird abundance from across a whole mountain chain in Northern Europe to (1) quantify the overall speed and extent of altitudinal shift in birds’ abundance over the past decade under climate change, and (2) assess whether species’ altitudinal abundance shifts are related to their traits. Given the high rates of climate warming in the study region (IPCC 2014) and in mountain areas more generally (Thompson 2000; Brunetti et al. 2009), we expect that the mean altitude of the bird species’ abundance shifted uphill during the study period and this shift being faster in areas with higher altitudinal space. We also expect species-specific altitudinal abundance shifts to vary along four trait gradients (Laiolo and Obeso 2017): (1) fastness–slowness of species’ life history (body mass, clutch size, and longevity), (2) ecological niche (habitat association, diet specialization, and climatic niche), (3) migration behaviour (migration strategy), and (4) population dynamics (population trend). The variables above have commonly been used in species’ range and abundance shift and climate change analyses (Devictor et al. 2008; MacLean and Beissinger 2017; Tayleur et al. 2016). We expect species with faster life histories and wider habitat niche to respond more rapidly to changing climatic conditions (as reported by Välimäki et al. 2016), thus showing faster uphill shifts in abundance. Moreover, we expect resident and short-distance migrant species to respond faster, thus showing more pronounced uphill shifts as they overwinter at higher latitudes where climate change is most rapid (Auer and King 2014; Välimäki et al. 2016). We expect a larger shift in mean altitude of abundance for species with preference for colder climatic niches, as they may be more forced to seek optimal cooler conditions (Tayleur et al. 2016). Finally, abundance of species with positive population trend is expected to shift faster uphill, because more individuals are available for colonizing higher-altitude areas (Koschová and Reif 2014, Ralston et al. 2017; Flousek et al. 2015).

## Materials and methods

### Data

**Fig. 1:**
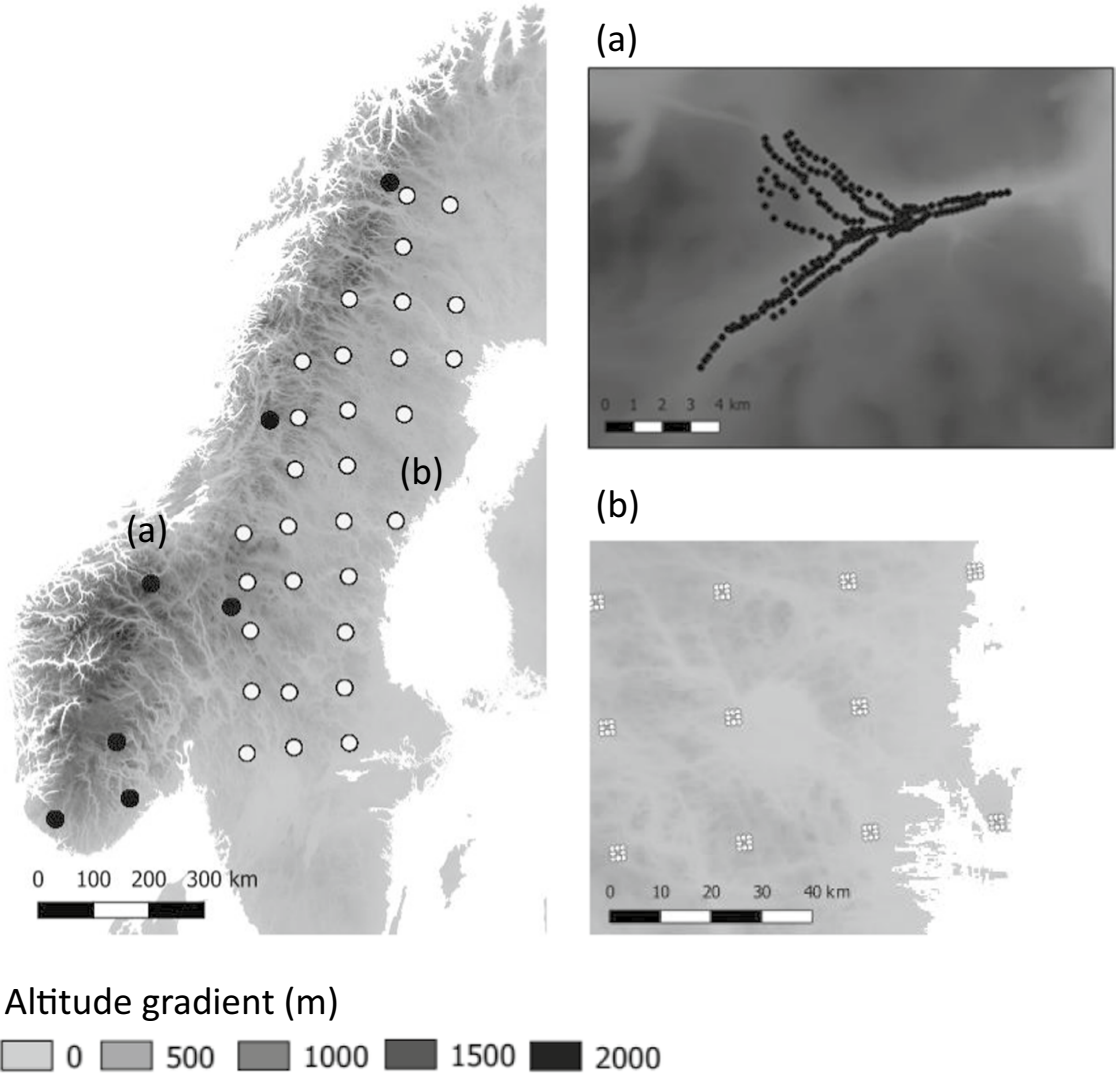
The locations of the geographical centroids of the grid cells included in the analyses. Black dots represent Norwegian centroids of grid cells and white dots represent Swedish centroids of grid cells. Altitude of the study locations varied between 325 and 1225 m. Maps (**a**) and (**b**) show examples of study design within grid cells in Norway and in Sweden, respectively. Each dot on maps (**a**) and (**b**) represents a surveyed point within a route. The altitude information is based on European Union, Copernicus Land Monitoring Service (2020)

We used species and topographic data from the Scandinavian mountains (Fig. 1). We obtained the altitudinal information at 25 m resolution (European Union, Copernicus Land Monitoring Service 2020) for all survey points using QGIS software (version 3.4.14.). In addition, we obtained the monthly temperature data for all survey points. Data from all weather stations in Sweden (approximately 300 stations spread evenly across the country) have been interpolated to a 4 × 4 km grid, using geo-statistic interpolation (Johansson 2000). For Norway, weather data were available from Norwegian observational gridded climate datasets available at https://thredds.met.no/thredds/catalog/senorge/seNorge_2018/Archive/catalog.html. For the analyses, we selected the interpolated weather data closest to each mountainous site.

We obtained bird abundance data from two monitoring schemes carried out in Norway and Sweden. The Norwegian data span from 1990 to present, with seven survey locations distributed across the country to cover a wide range of climatic conditions. Each survey location has 200 survey points situated along eight to ten survey routes each with 20–25 survey points. The distance between the survey points within a survey route is 200–300 m (Solbu et al. 2018) (Fig. 1a), totalling 1400 point counts across an altitudinal range from 200 to 1350 m. At each point count location, a five-minute recording of all birds seen and heard was carried out yearly from late May to early July (one day visit per year). The Swedish data follow the “fixed routes” (Lindström et al. 2013), and span from 1996 to present. A total of 716 routes are distributed across the country and across a 25 km grid. Each route consists of an eight km line transect that forms a 2 * 2 km square (Fig. 1b), which includes eight point count locations (one per km). Thus, the total number of surveyed points is 5728. The altitude varies from 0 to 1207 m. The surveys were carried out yearly from mid-May (southern Sweden) to early July (northern Sweden). All individual birds seen and heard during a survey were recorded.

We used eight species’ traits as explanatory variables in the analysis of the role of species-specific differences in altitudinal abundance shifts: clutch size (Storchová and Hořák 2018), longevity (De Magalhaes and Costa 2009), body mass (Wilman et al. 2014), main habitat (Lehikoinen and Virkkala 2016), diet specialization (modified from Wilman et al. 2014), migration strategy (Laaksonen and Lehikoinen 2013), species thermal index (STI, Devictor et al. 2008), and population trend (Green et al. 2019) (for more details, see Table S1).

### Data selection

We investigated altitudinal shifts that occurred between two four-year study periods (period 1: 1999–2002 and period 2: 2015–2018), for which there are adequate data for both countries. Compiling data into study periods of four years allows reducing the potential effect of random environmental stochasticity among monitoring seasons. We selected survey points that have been surveyed in at least one year during both study periods. In total, there were 4835 survey points in common between study periods (1400 Norwegian points and 3435 Swedish points). We divided the selected survey points into grid cells. For the Norwegian data, we used the location of the seven survey areas as they were distant from each other (Fig. 1). For the Swedish data, we divided the country into grid cells of 100 * 100 km^2^. Since we were interested in the altitudinal abundance shifts, we included only those grid cells that comprised a minimum altitudinal range (i.e., difference between the minimum and the maximum altitude) of 300 m and contained at least 10 survey points. Consequently, we excluded from the analyses the lowlands of Sweden and focused on the more hilly regions, in addition to the Scandinavian mountain range that runs along the Swedish–Norwegian border (Fig. 1). In total, there were seven grid cells in Norway, where each grid cell had 200 survey points. In Sweden, there were 30 grid cells with 13 to 118 survey points (Fig. 1).

Because we were interested in the general pattern of birds’ altitudinal abundance shifts, we removed observations of very rare species to ensure reliable estimates of shift speed. That is, we only included species that were observed in at least three grid cells. In addition, we calculated the mean annual number of individuals in each survey point in each study period and included in the analyses only those species for which the mean was at least five within a grid cell and study period. We excluded the non-native Canada goose (*Branta canadensis*) from the analyses, because its range expansion is not necessarily driven by climatic factors but by human-induced introduction programs. Overall, we included 76 bird species in the analyses (for the full list of species, see Table S2). To validate the robustness of the species selection procedure, we repeated the analyses with varying selection thresholds and did not find any major differences in the speed of species' abundance shift (Table S3).

### Statistical analyses

To assess the altitudinal abundance shift, we estimated the mean altitude of each species abundance for each grid cell and each study period in four steps.

(1) We calculated the average number of individuals (*A*) of a species per survey point per period:

$$A = \frac{{\sum {{\text{observations}}} }}{N}$$

where *N* is the number of years the point was surveyed in a given period.

(2) We created an altitudinal gradient for each grid cell from 300 to 1400 m by 50 m intervals and aggregated the average number of observations along this gradient.

(3) We estimated the mean abundance *R* of species *i* in each altitudinal interval of each grid cell:

$$Ri = \frac{\sum A }{{np}}$$

where *np* is the number of points inside the altitudinal interval of the grid cell.

(4) We estimated the mean altitude of each species (*M*_alt_) in a grid cell and period:

$$M_{{{\text{alt}}}} = \sum {\frac{Ri}{{\sum {Ri} }}} *Mi$$

where $\frac{Ri}{{\sum {Ri} }}$ is the mean abundance of a species in each grid cell and *Mi* the mean altitude of the survey points within an altitudinal interval of a grid cell.

We performed a linear mixed effects model (Gaussian distribution with identity link) to test for changes in the mean altitude of the 76 study species’ abundances between the two study periods using packages *lme4* (Bates et al. 2015) and *lmerTest* (Kuznetsova et al. 2017) in R software (version 4.0.5, R Core Team 2020). The response variable was the mean altitude of species’ abundance in each period and grid cell (*N* = 1812 species and grid combination). First, we investigated if there has been any general change in the mean altitude of species’ abundance. To account for the effects of topography and environment on abundance shifts, we included three topographical and spatio-temporal variables as fixed effects in the model: the study period (as a continuous variable: period 1 and 2), the altitudinal range within the survey sites inside the grid, and the mean longitude of the grid cell. We excluded latitude due to its strong correlation with longitude (*N* = 37, r_s_ = 0.739, *p* < 0.001) and altitudinal range (*N* = 37, r_s_ = 0.408, *p* = 0.010). Inclusion of longitude allowed us to account for potential spatial autocorrelation. We included the identities of country, grid cell, and species as random factors.

Second, we wanted to investigate whether potential altitudinal shifts were affected by the interaction of the study period with the longitude and altitudinal range of the grid cell. For this, we used the above-mentioned model and added the interactions between period and longitude, and period and altitudinal range.

To validate the robustness of the model results we first visually inspected spatial correlograms of the model residuals for a maximal distance of 500 km using package *ncf* (Bjornstad 2020) for R software. We found no sign of spatial autocorrelation in the residuals at any distance (Figure S1). Second, we fitted the same linear mixed model with different data selection criteria (e.g. by varying selection criteria for number of grid cells, number of species, and number of individuals; Table S3). Moreover, we confirmed the temperature trend across the study area using the grid cell-specific monthly temperatures from Sweden and Norway to calculate the mean temperature for each study period. More specifically, we averaged the monthly temperatures of March and April (early spring), May and June (late spring – early summer) and July—August (late summer) in each year and then averaged those yearly means across the years within the study period. We used paired t test (function t.test in R program) to quantify the temperature change between the two periods.

To identify those species’ traits that may drive the speed of the altitudinal shift, we considered as a response variable the calculated average species-specific abundance change in mean altitude between periods across grid cells, and as explanatory variables species’ traits. We excluded common raven (*Corvus corax*) from this analysis because its longevity trait value was recorded for a captive individual, whereas longevity trait values of other species were recorded for wild individuals. Due to the strong correlation between body mass and both clutch size (*N* = 75, r_s_ = -0.440, *p* < 0.001) and longevity (*N* = 75, r_s_ = 0.616, *p* < 0.001), we excluded body mass from the main analysis, but we also reran the analyses where the longevity was replaced by body mass. The other variables did not show strong collinearity (|r|< 0.50). Before fitting the models, we standardized all continuous explanatory variables to zero mean and unit SD to aid computation and facilitate comparison of the effect sizes among the different trait variables. Because closely related species can have similar responses and similar traits, we explicitly accounted for the phylogenetic structure in the models. We first obtained a consensus tree from 100 phylogenetic trees downloaded from birdtree.org (Jetz et al. 2012) using the function consensus (package ape; Paradis and Schliep 2019) in R. Then, we fitted phylogenetic generalized linear models using the function pgls from package caper (Orme et al. 2018) in R software to test all the possible combinations of hypotheses, totalling 16 models (Table 3). We performed model selection based on Akaike information criterion for small sample sizes (AICc) using package *MuMIn* in R software (Barton, 2019). In the case of several equally well supported models, we performed model averaging of all the models within 4 AICc units (Burnham and Anderson, 2004).

The sampling design varies between the two study countries due to different sampling methods. Because of this, we reran the main analyses (altitudinal shifts and trait analyses) using only the Swedish data, which are more spatially structured. The results (not shown here) were qualitatively similar to those obtained using the whole data set.

## Results

### Temperature changes

The March–April were significantly higher (mean difference + 0.54 °C, t = − 8.01, df = 36, *P* < 0.001) across the study region during the second study period in, whereas May–June temperatures were significantly lower (mean difference − 0.25 °C, t = 2.17, df = 36, *P* = 0.037). The July–August temperatures did not show no temporal trend (difference − 0.04 °C, t = − 0.62, df = 36, *P* < 0.538).

### Shift in the mean altitude

**Fig. 2:**
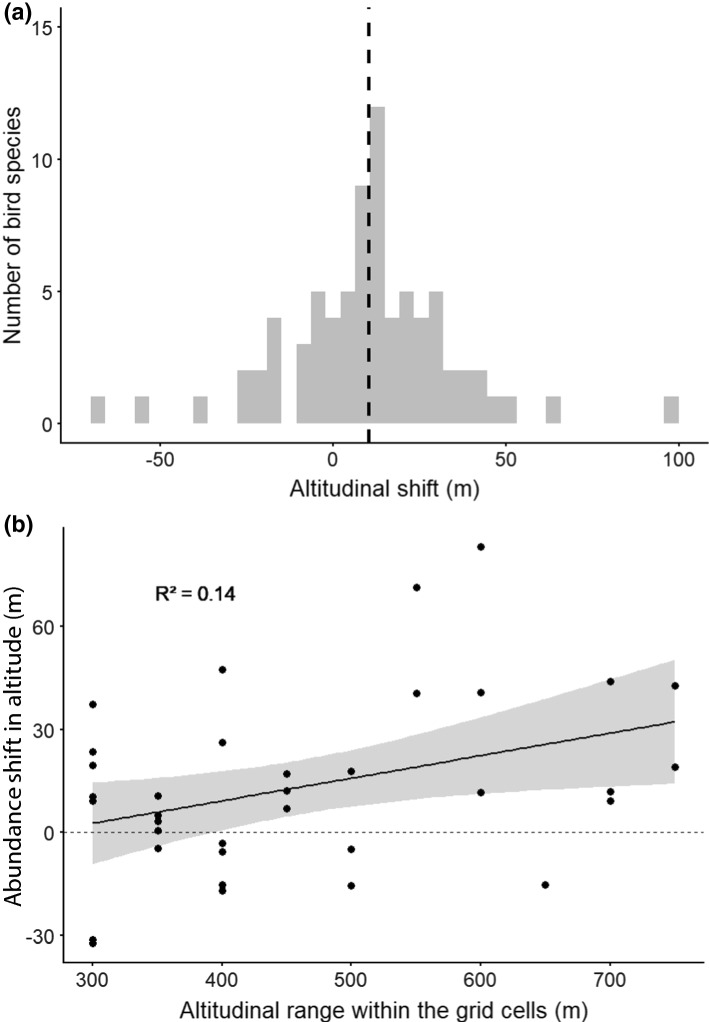
Distribution of the speed of altitudinal shift among 77 bird species between study periods (1999–2002 and 2015–2018). Panel **a** illustrates the number of species per altitudinal shift bin (y-axis). The speed of the altitudinal shift is shown on the x-axis such that the negative values indicate downhill shift and the positive values indicate uphill shift. Dashed vertical line corresponds to the average altitudinal shift across the species. The values are obtained from the raw data. Panel **b** illustrates the relationship between the average altitudinal shift across bird species and the altitudinal range within grid cells. Each black dot represents one grid cell. Black line represents the linear regression relationship of the variables, while the dark grey area represents the 95% confidence interval. The average speed of the altitudinal shift across species is shown on the y-axis such that the negative values indicate an average downhill shift and the positive values indicate an average uphill shift in the bird community within the grid cell. Altitudinal range within the grid cell, shown on the x-axis, is measured as the difference between the minimum and the maximum altitude of any location within the grid cell

According to our first altitudinal shift model, bird abundance moved uphill on average by 12.3 m from 1999–2002 to 2015–2018 (Fig. 2a, Table 1). This corresponds approximately to 0.9 m per year. Of the 76 species, 7 showed a significant uphill abundance shift (altogether 54 species had slope towards uphill), while 3 species shifted significantly downhill (22 species had slope towards downhill) (Table S4). The overall mean altitude of bird abundance was higher in the west and in grid cells with a larger altitudinal range (Table 1). Our interaction model showed that altitudinal abundance shifts correlated to the topography of the grid, being faster in grid cells with a larger altitudinal range (Table 2, Fig. 2b, Fig. S3).

**Table 1:** Results of mixed model analysis on the mean altitude of bird species abundance

Variable	Estimate	SE	Df	*t* value	*p* value
Intercept	389.23	127.69	15.8	3.05	0.008
Period	12.29	2.92	1696.7	4.21	< 0.001
Mean longitude	− 18.65	6.50	27.2	− 2.87	0.008
Altitudinal range	0.81	0.15	33.5	5.36	< 0.001

Period refers to the categorical variable, whereby the statistics relate to the most recent study period (2015–2018), and the more distant study period (1999–2002) is set as the reference study period. Mean longitude is mean longitude of the grid and altitudinal range is altitudinal range of the grid

**Table 2:** Results of mixed model analysis on the mean altitude of bird species including interaction between period and spatial variables: study period, mean longitude of the grid and altitudinal range of the grid

Variable	Estimate	SE	Df	*t* value	*p* value
Intercept	413.70	129.79	16.89	3.19	0.005
Period	− 4.01	15.77	1694.75	− 0.25	0.779
Mean longitude	− 18.21	6.61	28.99	− 2.76	0.009
Altitudinal range	0.74	0.15	36.82	4.78	< 0.001
Period * Mean longitude	− 0.29	0.78	1694.75	− 0.376	0.707
Period * Altitudinal range	0.05	0.02	1694.75	2.12	0.034

Period refers to the categorical variable, whereby the statistics relate to the most recent study period (2015–2018), and the more distant study period (1999–2002) is set as the reference study period

### Role of species’ traits

**Fig. 3:**
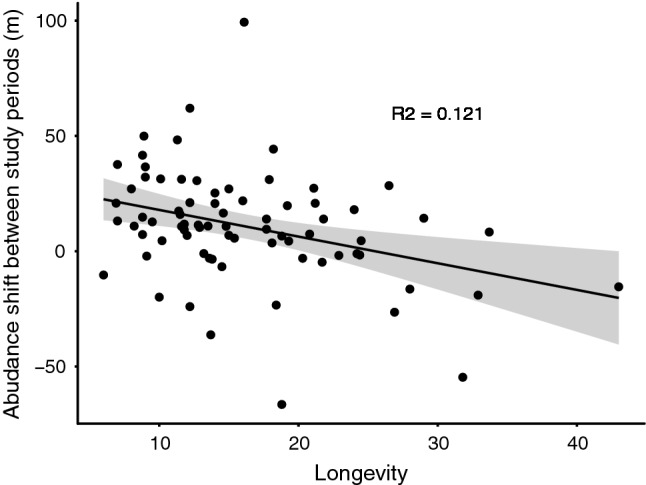
Relationship of the abundance shift between study periods (m) (1999–2002 and 2015–2018) and species’ longevity in years. Each black dot represents one species (*N* = 75). Fitted line represents the least square regression line and dark grey area is the 95% confidence interval. The explanatory power of the linear relationship is shown within the panel

The model selection procedure identified two best-supported models explaining the species-specific speed in altitudinal abundance shift (Table 3). After averaging these two models, the mean altitudinal shift of birds’ abundance was best explained by the fastness–slowness life-history continuum (Table 4), whereby short-lived species showed significantly faster uphill shifts in abundance compared to long-lived species (Fig. 3). None of the other tested traits, including body mass, were significantly related to the mean altitudinal abundance shift (Table 4, Table S5).

**Table 3:** Summary of model selection showing **∆**AICc values of linear mixed effects models explaining variation in the extent of the altitudinal abundance shift according to the species’ trait hypotheses tested

Hypothesis	Explanatory variables	∆AICc
Fastness-slowness + population dynamics	Clutch size + Longevity + Population trend	0
Fastness-slowness	Clutch size + Longevity	0.33
Fastness-slowness + migratory behaviour + population dynamics	Clutch size + Longevity + Migration strategy + Population trend	1.12
Fastness-slowness + migratory behaviour	Clutch size + Longevity + Migration strategy	1.64
Migratory behaviour	Migration strategy	5.80
Migratory behaviour + population dynamics	Migration strategy + Population trend	5.81
Null model	Null	6.50
Population dynamics	Population trend	6.87
Fastness–slowness + Ecological niche	Clutch size + Longevity + Main habitat + Diet specialization + STI	7.04
Fastness–slowness + ecological niche + population dynamics	Clutch size + Longevity + Main habitat + Diet specialization + Population trend + STI	9.05
Fastness–slowness + ecological niche + migratory behaviour	Clutch size + Longevity + Main habitat + Diet specialization + STI + Migration strategy	10.65
Ecological niche	Main habitat + Diet specialization + STI	11.76
Full model	Clutch size + Longevity + Main habitat + Diet specialization + Migration strategy + Population trend + STI	12.55
Ecological niche + population dynamics	Main habitat + Diet specialization + STI + Population trend	13.92
Ecological niche + migratory behaviour	Main habitat + Diet specialization + STI + Migration strategy	14.20
Ecological niche + migratory behaviour + population dynamics	Main habitat + Diet specialization + Migration strategy + Population trend + STI	16.25

The rows are ordered according to the increasing **∆**AICc values

**Table 4:** Results of the averaged models of altitudinal abundance shift as a function of species’ traits

Variable	Estimate	Standard error	*t* value	*p* value
Intercept	30.16	11.49	2.62	0.009
Clutch size	− 3.95	3.30	1.19	0.231
Longevity	− 1.32	0.41	3.19	0.001
Population trend	2.69	3.22	0.81	0.419
Migration strategy, partial	− 0.96	6.23	0.16	0.877
Migration strategy, SDM	− 6.20	9.96	0.62	0.534
Migration strategy, LDM	− 3.71	7.38	0.50	0.616

The best models within four AICc units (Table 3) were averaged, i.e. restricting the explanatory variables

*Partial* partial migrant, *SDM* short-distance migrant, *LDM* long-distance migrant

## Discussion

We found that bird species’ abundances across and around the Scandinavian mountains shifted uphill over the past decade. Moreover, we showed that the magnitude of the altitudinal abundance shift is uneven in space and when considering species’ traits, best explained by longevity. Through this study period, the early spring temperatures have increased, quite substantially, whereas the late spring – early summer temperatures have decreased, but to a smaller degree. The late summer temperatures did not show clear trend during the study period. Despite the recent contrasting trends in the temperature depending on the season, the temperature in recent decades is significantly warmer than a couple of decades ago (Lehikoinen et al. 2014), which reflects the overall long-term increase in temperature in North Europe since the 1960s (European Environment Agency 2017). This could indicate warming is a candidate factor driving the observed altitudinal shifts in bird abundances. However we do not know the exact population dynamical mechanisms including lag effects how the temperatures or other weather variables such as snow conditions could contribute to the altitudinal shifts of species.

The mean altitude of bird species’ abundances has moved uphill at an average speed of 0.95 m per year, which is a similar rate of change as that of distribution shifts reported in a meta-analysis (1.1 m/year) by Chen et al. (2011). This suggests that abundance shifts are not only driven by a small number of individuals at the range boundaries, but the overall bird abundances are on the move. The observed uphill shift aligns with our expectations under increased early spring temperatures in the study region (IPCC 2014), and with earlier studies reporting bird distributions to be sensitive to temperatures (Böhning-Gaese and Lemoine 2004). The observed uphill shifts in abundances are also in line with earlier studies using presence–absence data that have documented uphill shifts in Southeast Asia (Peh 2007), North America (DeLuca and King 2017), and Europe (Maggini et al. 2011; Reif and Flousek 2012).

Our findings also illustrate that abundance shifts along elevational gradients are faster in areas that have a wider altitudinal range and our sensitivity analyses using various data selection criteria indicate that the results are robust. These areas of high altitudinal heterogeneity may also hold more space available uphill, which may in turn facilitate a more rapid shift of species abundance. This suggests, at least partly, that birds' abundance shifts uphill may be limited, to some extent, by the topography of the landscape (Elsen et al. 2020).

Overall higher number species shifted their abundance uphill than downhill. Importantly, we found that short-lived species shifted their abundance towards higher grounds more than long-lived species. Longevity is strongly associated with reproduction rate (Angert et al. 2011). Thus, long-generation lengths may cause the species to have a lower potential for responding fast to changing circumstances and subsequently lead to limited altitudinal or latitudinal shifts compared to short-lived species (Brommer 2008; Auer and King 2014; Välimäki et al. 2016; Böhm et al. 2016; Pacifici et al. 2020). Our result can be interpreted in two ways. On the one hand, species with a slow turnover of generations can be more vulnerable to climate change (Foden and Young 2016), because they are less capable of rapidly responding to climate change by shifting to higher altitudes or latitudes (Brommer 2008; Auer and King 2014; Välimäki et al. 2016). Furthermore, species with slow life histories are also more extinction prone (Sodhi et al. 2009). The high extinction risk of slow-reproducing species is also related to their particular niche properties: slow species typically occur at low densities and require larger areas that may become a limited resource under climate change, particularly in mountain areas where space shrinks further up. Thus, on top of all other drivers of extinction risk, climate change may exert a particularly strong pressure on long-lived mountain species.

Conversely, the finding that short-living species seem to be more capable of shifting towards higher altitudes (as shown in our study) or latitudes (e.g. Välimäki et al. 2016) may suggest that these species are more capable of coping with change. In practice, however, several of such species may be already declining. The common mountain bird monitoring in Fennoscandia has shown that high altitude passerines, like snow bunting (*Plectrophenax nivalis*) and Lapland bunting (*Calcarius lapponicus*), are already declining (Lehikoinen et al. 2019). Thus, the vulnerability of a mountain species is not necessarily dependent on its abundance shift speed, but also on the mean altitude at which it occurs, with higher-altitude species possibly being more threatened. Based on our results, cold-dwelling passerines, such as bluethroat (*Luscinia svecica*), northern wheatear (*Oenanthe oenanthe*) and common redpoll (*Acanthis flammea*), show faster altitudinal shifts than all species on average (Table S4) and may be under higher threat.

Beyond longevity, species’ traits did not significantly affect altitudinal abundance shifts. This might be explained by the strong link between species’ need to find suitable climatic conditions and their capacity to colonise new areas (Angert et al. 2011). Indeed, as the rate of warming is particularly high in the study area (Thompson 2000; Brunetti et al. 2009; IPCC 2014), species may move uphill to cooler habitats to survive, even if it means reaching the limit of some other axes of their niche, such as food- or nesting-site resources, which are linked to traits like species’ habitat and diet, e.g. forest birds reaching the tree-line.

Contrary to our expectation, 22 of the 76 species shifted their ranges downhill (Table S4). This may have multiple causes, such as sink-source dynamics (e.g. declining curlew population *Numenius arquata*; Lindström et al. 2019), competition dynamics, or random variation in fluctuation in food resources, such as rodents (e.g. in rough-legged buzzard *Buteo lagopus*) or seeds of trees (Gallego Zamorano et al. 2018; Sundell et al. 2004). Indeed, competition for resources may represent a plausible driver of the contrasting uphill and downhill shifts in species’ abundance reported here. The land area, and supposedly the amount and diversity of resources, including the carrying capacity of the environment, shrink towards higher altitudes (Laiolo et al. 2005). As a result, interspecific competition for these shrinking resources could increase if all species simultaneously move uphill. Thus, it is somewhat predictable that, under resource limitations, species might show opposite patterns of abundance shifts to minimise competition.

At a general level, species may respond to climate change in three ways: shift their range or abundance in search for climatically optimal areas, adapt locally for example via shifts in phenology, or decline in numbers and eventually go extinct locally or globally (Parmesan 2006; Hällfors et al. 2021). Typically, species that have been shown to shift fastest under climate change have been classified as winners, and those shifting slowest as losers (e.g., Tayleur et al. 2016). However, in mountain areas, the amount of suitable habitat typically shrinks towards higher altitudes. Therefore, species that are shifting faster, and potentially occupy already high altitudes at present, may in fact appear as winners at present, but become losers in the long run. This is because such rapidly shifting species, such as the short-lived species in our study, when occurring at already high altitudes, may face much faster reductions in the overall range, as shifts in the leading altitudinal range edge will inevitably be hindered by physical constraints, such as by reaching the mountaintop (Elsen et al. 2020). Conversely, species shifting slower will take longer to reach the mountaintop and thus may preserve their range extent for longer. However, due to climate change and warming spring temperatures in the study region (European Environment Agency 2017), such species will persist in potentially suboptimal climatic niches, which may hamper their survival and reproduction. Ultimately, assessing the relative importance of stressors, such as human-induced range and abundance shifts, and species’ adaptation capacity, will be key to assessing population persistence under climate change and identify losers and winners at present, but also in the near and far future.

The altitudinal shifts are complex because they can depend on various underlying processes. In our study, we observed clear shifts uphill despite only early spring temperatures having increased. Importantly, microclimatic conditions and sun exposure differ strongly between slopes, particularly between northern and southern slopes, and may be mediated by the land cover type. Similarly, changes in land use and in species’ interactions may be important in further explaining altitudinal range shifts (Heikkinen et al. 2007; Bateman et al. 2016; Reino et al. 2018). For example, the temperature-driven changes in availability of prey species may affect breeding success of predator species and lead into species-specific changes in the speed of range shift (Pearce-Higgins et al. 2010; Pearce-Higgins and Green 2014). It is still poorly understood how these different abiotic and biotic factors interact in affecting the speed of altitudinal abundance shifts. Furthermore, understanding the complex microclimatic effects on range and abundance shifts is important for conservation as the colder northern slopes could serve as microclimatic refugia for cold-dwelling species. Therefore, future research should assess the fine-scale differences in altitudinal shifts to understand the role of microclimate in the shift speed. Similarly, future research should aim at disentangling the independent and joint effects of climate versus land-use change in driving mountain bird abundance and range shifts. The land-use change was not accounted for in this study.

Ultimately, our results emphasize that altitudinal shifts are occurring at large spatial scales and affect species differently, with long-lived species showing the weakest responses. These results are thus particularly important for facilitating future assessments of species vulnerability to climate change, and to furthering our understanding on species’ adaptation and persistence under global change.

## Supplementary Information

Below is the link to the electronic supplementary material.

Supplementary file1 (DOCX 218 KB)

## Data Availability

The data are available upon request.

## Code availability

The code of the analyses is available upon a request.
