# Supplementary material for: Short-lived species move uphill faster under climate change
Source: Oecologia. 2022 Jan 6;198(4):877–88. doi: 10.1007/s00442-021-05094-4 (PMC9056483; doi:10.1007/s00442-021-05094-4)
Supplement: Supplementary file 1 — Supplementary file1 (DOCX 218 KB) [file 442_2021_5094_MOESM1_ESM.docx]

**Supplementary material**

**Appendix S1**

**Couet et al.**

**Short-lived species move uphill faster under climate change**


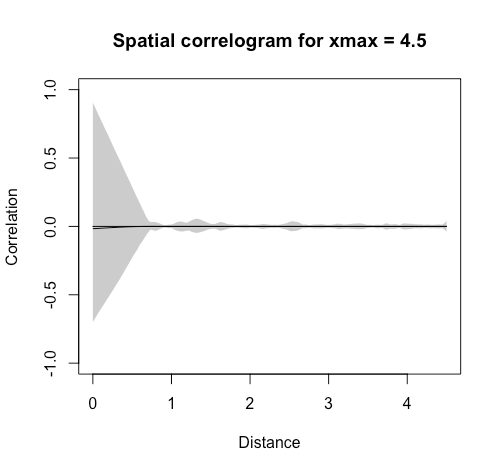


**Figure S1.** Spatial autocorrelation of the linear mixed model residuals of the relationship between the average altitudinal abundance shift across bird species and the geographical and spatial variables. Spatial correlogram illustrates the spatial autocorrelation of the residuals from the model for a maximal distance of 4.5 degree decimal, which corresponds to 500 km of distance.


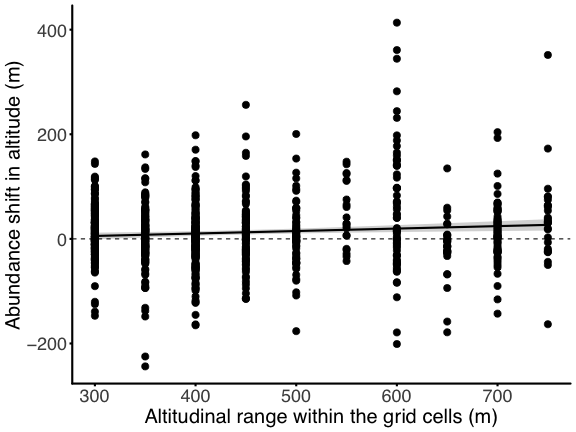


**Figure S2.** Altitudinal abundance shift of species within grid cells as a function of the altitudinal range of the grid. One dot is one species in a one grid cell.

**Table S1.** Definitions and references of each species’ trait used for the analysis of the role of species-specific differences in the altitudinal range shifts. References to data, trait definition, and computation tool origin are included. For full references, see the main text.

| **Trait** | **Definition** | **Reference(s)** |
| --- | --- | --- |
| Clutch size | Annual mean number of eggs | Storchová & Hořák 2018 |
| Longevity | Mean of maximum longevity (years) | De Magalhaes & Costa 2009 |
| Body mass | Mean body mass (g) | Wilman et al. 2014 |
| Main habitat | Main habitat that the species uses, divided into four categories: farmland-urban, forest, wetland and rocky outcrops, montane | Lehikoinen & Virkkala 2016 |
| Diet specialization | Quantified as Shannon’s diversity index (calculated with R package ‘vegan’, Oksanen *et al.*, 2019) of the proportional use of different diet categories: invertebrate, vertebrate (endotherm), vertebrate (ectotherm), fish, vertebrate (unknown), scavenge, fruit, nectar or pollen, seed, other plant material. | Wilman et al. 2014 |
| Migration strategy | Migration behaviour divided into four categories: resident, partial migrant (including species from which a part of the population migrates), short distance migrant (including species that winter in the Baltic countries or Western Europe), long distance migrant (including wintering further than the Mediterranean area) | Laaksonen & Lehikoinen 2013 |
| Species temperature index | Long-term average temperature experienced by individuals across the range (species temperature index, STI). The values were obtained from the combination of the spatial distribution of the mean temperature (WordlClim v. 1.0) of birds breeding season (from March to August) and data from the EBCC atlas of European breeding birds. STI was strongly correlated with mean altitude of species (r = -0.58), but it is independent measure of species climatic preference from our dataset. | Devictor et al. 2008, Hijmans et al. 2015, Hagemeijer & Blair 1997 |
| Population trend | The population tendency in the study area during the breeding season between the period 1998-2019, divided into three categories: decreasing population, stable population, increasing population. | Green et al. 2019 |

**Table S2.** List of species and their traits included in the analyses. For migration strategy (Migr.), the levels are: 1 = resident, 2 = partial migrant (species from which a part of the population migrates), 3 = short distance migrant (wintering in the Baltic countries or Western Europe), and 4 = long distance migrant (wintering further than the Mediterranean area). For the habitat, the levels are: 1 = farmland-urban, 2 = forest, 3 = wetlands (rocky outcrops), and 4 = montane. Finally, for the population tendency (trend), the levels are: 1= decreasing, 2 = no trend, and 3= increasing population.

| Species | Common  name | Body  mass | Diet | Migr. | Habitat | STI | Pop.  trend | Clutch  size | Longevity |
| --- | --- | --- | --- | --- | --- | --- | --- | --- | --- |
| *Acanthis flammea* | Common  redpoll | 13 | 1.089 | 2 | 2 | 8.19 | 1 | 5 | 12.2 |
| *Actitis hypoleucos* | Common  sandpiper | 48 | 1.221 | 4 | 3 | 11.21 | 1 | 4 | 14.5 |
| *Anthus pratensis* | Meadow  pipit | 18.4 | 0.500 | 3 | 4 | 9.95 | 2 | 4 | 8.8 |
| *Anthus trivialis* | Tree  pipit | 23.33 | 1.228 | 4 | 2 | 11.24 | 3 | 4 | 8.8 |
| *Apus apus* | Swift | 37.6 | 0.000 | 4 | 1 | 12.45 | 1 | 2.5 | 21.1 |
| *Bucephala clangula* | Goldeneye | 918.56 | 0.940 | 3 | 3 | 8.47 | 1 | 9.5 | 18.4 |
| *Buteo lagopus* | Rough-legged  buzzard | 949.76 | 0.639 | 3 | 2 | 5.23 | 2 | 3.5 | 18.8 |
| *Calcarius lapponicus* | Lapland  bunting | 27.84 | 0.693 | 3 | 4 | 4.06 | 1 | 5.5 | 6 |
| *Certhia familiaris* | Treecreeper | 9 | 0.673 | 2 | 2 | 11.12 | 3 | 5.5 | 8.2 |
| *Chloris chloris* | Greenfinch | 26 | 0.000 | 2 | 1 | 12.60 | 1 | 5 | 13.6 |
| *Chroicocephalus*  *ridibundus* | Black-headed  Gull | 284 | 0.802 | 2 | 3 | 11.07 | 1 | 2.5 | 32.9 |
| *Columba palumbus* | Common Wood  Pigeon | 490 | 1.089 | 3 | 1 | 12.22 | 3 | 1.5 | 17.7 |
| *Corvus corax* | Raven | 927.97 | 2.025 | 1 | 2 | 11.74 | 2 | 5 | 69 |
| *Corvus cornix* | Hooded  Crow | 570 | 1.557 | 1 | 1 | 12.13 | 1 | 4.333 | 16.8 |
| *Corvus corone* | Carrion  Crow | 570 | 1.557 | 2 | 1 | 12.13 | 1 | 4.5 | 19.2 |
| *Corvus monedula* | Jackdaw | 246 | 1.314 | 2 | 1 | 12.96 | 3 | 5 | 20.3 |
| *Cuculus canorus* | Common  Cuckoo | 111.36 | 0.325 | 4 | 2 | 12.14 | 3 | 9.2 | 12.9 |
| *Cyanistes caeruleus* | Blue tit | 13.3 | 1.221 | 1 | 2 | 12.74 | 3 | 11 | 14.6 |
| *Cygnus cygnus* | Whooper  Swan | 9349.99 | 0.000 | 3 | 3 | 6.27 | 3 | 4 | 26.5 |
| *Delichon urbicum* | House  Martin | 14.5 | 0.000 | 4 | 1 | 12.35 | 1 | 4 | 15 |
| *Dendrocopos major* | Great spotted  Woodpecker | 74.94 | 1.168 | 1 | 2 | 12.12 | 3 | 5.5 | 12.7 |
| *Dryocopus martius* | Black  Woodpecker | 321 | 0.000 | 1 | 2 | 11.12 | 1 | 5 | 14 |
| *Emberiza citrinella* | Yellowhammer | 29.7 | 0.898 | 2 | 1 | 11.55 | 1 | 4 | 13.2 |
| *Emberiza schoeniclus* | Reed  Bunting | 18.4 | 1.030 | 3 | 3 | 11.27 | 1 | 4.5 | 12.2 |
| *Erithacus rubecula* | Robin | 17.7 | 1.609 | 3 | 2 | 12.00 | 3 | 5 | 19.3 |
| *Ficedula hypoleuca* | Pied  Flycatcher | 13.79 | 0.000 | 4 | 2 | 10.43 | 2 | 6.5 | 15 |
| *Fringilla coelebs* | Chaffinch | 23.81 | 0.950 | 3 | 2 | 12.30 | 3 | 4.5 | 29 |
| *Fringilla montifringilla* | Brambling | 23.19 | 1.055 | 3 | 2 | 7.03 | 1 | 6 | 14.8 |
| *Gallinago gallinago* | Snipe | 112.94 | 0.639 | 3 | 3 | 10.27 | 3 | 4 | 18.2 |
| *Garrulus glandarius* | Jay | 159.46 | 1.280 | 1 | 2 | 12.47 | 2 | 6 | 17.9 |
| *Gavia arctica* | Black-throated  Diver | 2251.1 | 0.802 | 3 | 3 | 6.83 | 2 | 2 | 28 |
| *Grus grus* | Common  Crane | 5499.99 | 1.609 | 3 | 3 | 9.50 | 3 | 2 | 43 |
| *Hirundo rustica* | Swallow | 17.91 | 0.639 | 4 | 1 | 12.43 | 3 | 4.5 | 16 |
| *Lagopus lagopus* | Willow  Ptarmigan | 566.86 | 0.325 | 1 | 2 | 6.74 | 1 | 7.5 | 9 |
| *Lagopus muta* | Rock  Ptarmigan | 535.3 | 0.802 | 1 | 4 | 6.03 | 2 | 6.5 | 12 |
| *Larus canus* | Common  Gull | 412.53 | 1.194 | 3 | 3 | 8.57 | 2 | 3 | 33.7 |
| *Lophophanes cristatus* | Crested  Tit | 11.04 | 0.950 | 1 | 2 | 11.71 | 3 | 6.5 | 11.6 |
| *Loxia curvirostra* | Crossbill | 38.29 | 1.030 | 2 | 2 | 10.49 | 3 | 4 | 16.1 |
| *Luscinia svecica* | Bluethroat | 17.23 | 1.089 | 4 | 4 | 10.16 | 2 | 5.5 | 11.4 |
| *Lyrurus tetrix* | Black  Cock | 1068.66 | 0.940 | 1 | 2 | 9.00 | 2 | 8.5 | 12.2 |
| *Motacilla alba* | Pied  Wagtail | 23.93 | 0.000 | 3 | 3 | 11.99 | 1 | 5.5 | 13.7 |
| *Motacilla flava* | Yellow  Wagtail | 17.68 | 0.940 | 4 | 3 | 12.14 | 2 | 5 | 8.8 |
| *Muscicapa striata* | Spotted  Flycatcher | 15.9 | 0.500 | 4 | 2 | 12.15 | 3 | 5 | 11.8 |
| *Numenius arquata* | Curlew | 802.99 | 1.089 | 3 | 1 | 10.05 | 1 | 4 | 31.8 |
| *Numenius phaeopus* | Whimbrel | 364.57 | 1.089 | 4 | 3 | 5.89 | 2 | 4 | 24.2 |
| *Oenanthe oenanthe* | Wheatear | 25.39 | 0.802 | 4 | 3 | 11.62 | 2 | 5.5 | 10.1 |
| *Parus major* | Great  Tit | 16.25 | 1.471 | 1 | 2 | 12.34 | 3 | 10 | 15.4 |
| *Periparus ater* | Coa  Ttit | 9.2 | 1.471 | 1 | 2 | 11.91 | 2 | 8.5 | 9.5 |
| *Phoenicurus*  *phoenicurus* | Redstart | 14.59 | 0.500 | 4 | 2 | 11.31 | 3 | 6 | 10.2 |
| *Phylloscopus collybita* | Chiffchaff | 8.3 | 0.639 | 3 | 2 | 11.95 | 3 | 5.5 | 8 |
| *Phylloscopus sibilatrix* | Wood  Warbler | 9.2 | 0.500 | 4 | 2 | 11.38 | 2 | 6 | 10 |
| *Phylloscopus trochilus* | Willow  Warbler | 8.7 | 0.639 | 4 | 2 | 10.46 | 2 | 6 | 11.8 |
| *Pica pica* | Magpie | 217.48 | 1.748 | 1 | 1 | 12.16 | 2 | 6 | 21.7 |
| *Pluvialis apricaria* | Golden  Plover | 214 | 0.940 | 3 | 4 | 7.00 | 2 | 4 | 12.8 |
| *Poecile montanus* | Willow  Tit | 11.1 | 0.950 | 1 | 2 | 10.20 | 1 | 7.5 | 11.3 |
| *Prunella modularis* | Dunnock | 20.24 | 0.693 | 3 | 2 | 10.91 | 3 | 5 | 20.8 |
| *Regulus regulus* | Goldcrest | 5.54 | 0.000 | 2 | 2 | 10.81 | 1 | 10 | 7 |
| *Saxicola rubetra* | Whinchat | 16.6 | 0.802 | 4 | 1 | 11.30 | 1 | 5.5 | 6.9 |
| *Spinus spinus* | Siskin | 13.24 | 1.314 | 2 | 2 | 10.24 | 3 | 4 | 13.5 |
| *Stercorarius*  *longicaudus* | Long-tailed  Jaeger | 287.9 | 0.898 | 4 | 4 | 3.57 | 2 | 2 | 14 |
| *Sturnus vulgaris* | Starling | 77.14 | 1.696 | 3 | 1 | 11.80 | 1 | 5 | 22.9 |
| *Sylvia atricapilla* | Blackcap | 16.7 | 1.168 | 4 | 2 | 12.62 | 3 | 5 | 13.8 |
| *Sylvia borin* | Garden  Warbler | 18.2 | 0.943 | 4 | 2 | 11.32 | 3 | 4.5 | 24 |
| *Sylvia communis* | Greater  Whitethroat | 15.1 | 0.898 | 4 | 1 | 12.65 | 3 | 4.5 | 8.9 |
| *Sylvia curruca* | Lesser  Whitethroat | 11.44 | 1.089 | 4 | 2 | 11.64 | 1 | 5 | 9 |
| *Tringa glareola* | Wood  sandpiper | 62.05 | 0.000 | 4 | 3 | 7.67 | 2 | 4 | 11.6 |
| *Tringa nebularia* | Greenshank | 187 | 0.639 | 4 | 3 | 6.45 | 2 | 4 | 24.4 |
| *Tringa ochropus* | Green  Sandpiper | 71.4 | 0.802 | 4 | 2 | 9.88 | 3 | 4 | 11.5 |
| *Tringa totanus* | Redshank | 129 | 0.639 | 3 | 3 | 11.04 | 3 | 4 | 26.9 |
| *Troglodytes troglodytes* | Wren | 9.74 | 1.228 | 3 | 2 | 12.24 | 3 | 6.5 | 7 |
| *Turdus iliacus* | Redwing | 61.2 | 1.280 | 3 | 2 | 8.25 | 1 | 5 | 18.8 |
| *Turdus merula* | Blackbird | 102.73 | 1.221 | 2 | 2 | 12.58 | 3 | 4 | 21.8 |
| *Turdus philomelos* | Song  Thrush | 67.74 | 1.089 | 3 | 2 | 11.40 | 3 | 4 | 17.7 |
| *Turdus pilaris* | Fieldfare | 106 | 0.611 | 3 | 1 | 10.03 | 1 | 5.5 | 18.1 |
| *Turdus torquatus* | Ring  Ouzel | 109 | 1.314 | 3 | 4 | 9.03 | 3 | 4 | 9.1 |
| *Turdus viscivorus* | Mistle  Thrush | 117.37 | 1.332 | 3 | 2 | 11.92 | 3 | 4 | 21.2 |
| *Vanellus vanellus* | Lapwing | 218.37 | 0.000 | 3 | 1 | 11.53 | 1 | 4 | 24.5 |

**Table S3.** Results of the sensitivity mixed model analyses for different combinations of the data selection criteria: the minimum altitudinal range within the grid cell (Ar), the minimum relative abundance of the included species per grid cell (Rag), and the minimum number of grid cells where the included species occur (Nbg). Each sensitivity model was structured as follows: Mean altitude ~ Period + Grid longitude + Altitudinal range grid + (1|Species) + (1|Country/Grid identity) (in a syntax of lmer –function in R). For each variable, the slope is reported. Asterisks (*) represents the significance of the respective slope (*** p < 0.001; ** p < 0.01; * p < 0.05).

| **Combinations** | **Rag: 5**  **Ar: 300**  **Nbg: 1** | **Rag: 5**  **Ar: 300**  **Nbg: 3** | **Rag: 5**  **Ar: 300**  **Nbg: 5** | **Rag: 3**  **Ar: 300**  **Nbg: 1** | **Rag: 3**  **Ar: 300**  **Nbg: 3** | **Rag: 3**  **Ar: 300**  **Nbg: 5** | **Rag: 10**  **Ar: 300**  **Nbg: 1** | **Rag: 10**  **Ar: 300**  **Nbg: 3** |
| --- | --- | --- | --- | --- | --- | --- | --- | --- |
| **Number of**  **species** | 97 | 76 | 59 | 112 | 87 | 72 | 79 | 53 |
| **Number of**  **grids** | 37 | 37 | 37 | 37 | 37 | 37 | 36 | 36 |
| **Intercept** | 388.98** | 389.22** | 402.61** | 375.67* | 391.63** | 387.05** | 367.36* | 384.58* |
| **Study period** | 12.25*** | 12.29*** | 13.11*** | 12.30*** | 12.67*** | 13.35*** | 9.84** | 9.87* |
| **Mean longitude**  **of the grid cell** | -18.66** | -18.65 ** | -18.65** | -18.13* | -18.32* | -18.13* | -17.46* | -17.50* |
| **Altitudinal range**  **of the grid cell** | 0.81*** | 0.81*** | 0.81*** | 0.81*** | 0.80*** | 0.80*** | 0.83*** | 0.83* |

**Table S4.** Mean altitudes of species abundances during first and second study periods, mean shift across grid cells, and standard error of the shift. Shifts that differ from zero are bolded (95% c.i. do not cross zero).

| **Species** | **Mean.alt1** | **Mean.alt2** | **Shift** | **SE** |
| --- | --- | --- | --- | --- |
| Actitis_hypoleucos | 525.7 | 519.0 | -6.7 | 39.7 |
| Anthus_pratensis | 814.2 | 829.0 | 14.8 | 9.4 |
| Anthus_trivialis | 434.3 | 441.5 | 7.2 | 9.2 |
| Apus_apus | 126.8 | 154.1 | 27.3 | 15.3 |
| Bucephala_clangula | 391.9 | 368.5 | -23.4 | 73.8 |
| Buteo_lagopus | 798.6 | 732.1 | -66.5 | 58.1 |
| Calcarius_lapponicus | 766.7 | 756.4 | -10.3 | 27.4 |
| Carduelis_chloris | 93.5 | 90.5 | -3.1 | 4.7 |
| **Carduelis_flammea** | **548.1** | **610.1** | **62.0** | **17.2** |
| Carduelis_spinus | 433.4 | 444.3 | 10.9 | 13.9 |
| Certhia_familiaris | 242.4 | 253.3 | 10.9 | 27.7 |
| Columba_palumbus | 307.4 | 321.4 | 13.9 | 15.0 |
| Corvus_corax | 545.7 | 537.5 | -8.2 | 24.9 |
| Corvus_corone | 286.9 | 319.1 | 19.4 | 23.0 |
| Corvus_monedula | 96.6 | 93.5 | -3.1 | 19.4 |
| Cuculus_canorus | 508.6 | 518.9 | 10.3 | 11.2 |
| Cygnus_cygnus | 483.9 | 512.4 | 28.4 | 57.2 |
| Delichon_urbicum | 366.9 | 393.9 | 27.0 | 19.3 |
| Dendrocopos_major | 256.6 | 287.2 | 30.6 | 18.1 |
| Dryocopus_martius | 265.5 | 286.2 | 20.7 | 21.5 |
| Emberiza_citrinella | 152.7 | 151.7 | -1.0 | 35.3 |
| Emberiza_schoeniclus | 628.3 | 649.4 | 21.1 | 19.0 |
| Erithacus_rubecula | 409.8 | 414.2 | 4.4 | 8.8 |
| Ficedula_hypoleuca | 318.8 | 325.7 | 6.9 | 9.3 |
| **Fringilla_coelebs** | **408.3** | **422.6** | **14.3** | **5.8** |
| Fringilla_montifringilla | 534.0 | 544.8 | 10.8 | 5.9 |
| Gallinago_gallinago | 453.3 | 497.5 | 44.3 | 36.6 |
| Garrulus_glandarius | 232.5 | 263.6 | 31.1 | 37.0 |
| Gavia_arctica | 435.1 | 418.6 | -16.4 | 17.9 |
| Grus_grus | 215.3 | 199.9 | -15.5 | 24.8 |
| Hirundo_rustica | 141.0 | 162.8 | 21.8 | 20.9 |
| Lagopus_lagopus | 781.3 | 817.9 | 36.6 | 26.3 |
| Lagopus_muta | 999.8 | 1006.6 | 6.8 | 16.7 |
| Larus_canus | 454.2 | 462.5 | 8.3 | 26.4 |
| **Larus_ridibundus** | **123.7** | **104.6** | **-19.1** | **8.8** |
| **Loxia_curvirostra** | **289.4** | **388.7** | **99.3** | **41.5** |
| Luscinia_svecica | 759.4 | 776.8 | 17.4 | 13.4 |
| Motacilla_alba | 434.2 | 398.0 | -36.3 | 32.6 |
| **Motacilla_flava** | **541.8** | **583.4** | **41.6** | **19.3** |
| Muscicapa_striata | 387.5 | 396.7 | 9.2 | 17.2 |
| Numenius_arquata | 238.5 | 183.8 | -54.7 | 69.1 |
| Numenius_phaeopus | 538.7 | 537.6 | -1.1 | 29.6 |
| Oenanthe_oenanthe | 878.1 | 909.4 | 31.4 | 39.7 |
| Parus_ater | 221.2 | 233.9 | 12.7 | 35.8 |
| Parus_caeruleus | 124.0 | 140.5 | 16.5 | 18.2 |
| Parus_cristatus | 239.2 | 270.4 | 31.2 | 38.2 |
| Parus_major | 356.6 | 362.2 | 5.6 | 8.2 |
| **Parus_montanus** | **450.0** | **498.3** | **48.3** | **20.3** |
| Phoenicurus_phoenicurus | 493.5 | 498.0 | 4.5 | 5.2 |
| Phylloscopus_collybita | 243.2 | 270.3 | 27.0 | 14.1 |
| Phylloscopus_sibilatrix | 201.0 | 181.0 | -19.9 | 16.7 |
| **Phylloscopus_trochilus** | **483.0** | **494.7** | **11.7** | **5.9** |
| Pica_pica | 115.3 | 110.5 | -4.8 | 13.4 |
| Pluvialis_apricaria | 902.1 | 913.4 | 11.3 | 8.8 |
| Prunella_modularis | 431.9 | 439.3 | 7.4 | 13.3 |
| Regulus_regulus | 370.9 | 384.0 | 13.1 | 16.7 |
| Saxicola_rubetra | 246.9 | 267.7 | 20.8 | 22.0 |
| Stercorarius_longicaudus | 838.0 | 863.2 | 25.2 | 33.9 |
| Sturnus_vulgaris | 104.3 | 102.5 | -1.8 | 19.9 |
| Sylvia_atricapilla | 261.4 | 257.9 | -3.5 | 11.4 |
| Sylvia_borin | 214.9 | 232.9 | 18.0 | 14.1 |
| Sylvia_communis | 231.4 | 281.3 | 49.9 | 26.2 |
| Sylvia_curruca | 340.4 | 372.5 | 32.1 | 38.8 |
| **Tetrao_tetrix** | **404.9** | **380.9** | **-24.0** | **10.4** |
| Tringa_glareola | 526.5 | 537.2 | 10.7 | 20.0 |
| Tringa_nebularia | 515.1 | 513.5 | -1.7 | 23.1 |
| Tringa_ochropus | 273.2 | 289.1 | 15.9 | 29.9 |
| **Tringa_totanus** | **935.9** | **909.4** | **-26.5** | **6.4** |
| **Troglodytes_troglodytes** | **295.4** | **333.0** | **37.6** | **15.7** |
| Turdus_iliacus | 454.2 | 460.8 | 6.6 | 10.2 |
| Turdus_merula | 315.0 | 329.0 | 14.0 | 16.3 |
| Turdus_philomelos | 444.4 | 453.9 | 9.5 | 9.8 |
| Turdus_pilaris | 429.4 | 433.0 | 3.6 | 13.8 |
| Turdus_torquatus | 811.8 | 809.6 | -2.1 | 27.7 |
| Turdus_viscivorus | 374.4 | 395.2 | 20.8 | 20.6 |
| Vanellus_vanellus | 52.5 | 57.0 | 4.5 | 4.4 |

​​

**Table S5.** Additional trait analyses in models where longevity has been replaced with body mass. Number of parameters, AICc and AIC difference are shown. The null model is the same as in Table 3.

Model K ∆AIC

Null model  1 0.00

Body mass+Clutch size+Migration strategy    6 3.36

Body mass+Clutch size+Migration strategy+Population trend 7 3.64

Body mass+Clutch size  3 3.82

Body mass+Clutch size+Population trend  4 3.84

Body mass+Clutch size+Main habitat+Diet specialization+STI 8 9.73

Body mass+Clutch size+Migration strategy+Main habitat+

Diet specialization+STI 11 11.50

Body mass+Clutch size+Main habitat+Diet specialization+

STI+Population trend  9 12.21

Body mass+Clutch size+Migration strategy+Main habitat+

Diet specialization+STI+Population trend 12 14.17

**Supplementary references**

De Magalhaes JP, Costa J (2009) A database of vertebrate longevity records and their relation to other life‐history traits. Journal of Evolutionary Biology 22:1770–1774.

Devictor V, Julliard R, Couvet D, Jiguet F (2008) Birds are tracking climate warming, but not fast enough. Proceedings of the Royal Society B: Biological Sciences, 275:2743–2748.

Green M, Haas F, Lindström Å (2019) Monitoring population changes of birds in Sweden. Annual report for 2018. Department of Biology, Lund University, Lund. 92 pp.

Hagemeijer WJ, Blair MJ (1997) The EBCC atlas of European breeding birds. Poyser, London*.*

Hijmans RJ, Cameron SE, Parra JL, Jones PG, Jarvis A (2005) Very high resolution interpolated climate surfaces for global land areas. International Journal of Climatology: A Journal of the Royal Meteorological Society 25(15):1965–1978.

Laaksonen TK, Lehikoinen A (2013) Population trends in boreal birds: continuing declines in long-distance migrants, agricultural and northern species. Biological Conservation 168:99–107.

Oksanen J, Blanchet FG, Friendly M, Roeland K, Legendre P, McGlinn D, Minchin PR, O'Hara RB, Simpson GL, Solymos P, Stevens MHH, Szoecs E, Wagner H (2019) vegan: Community Ecology Package. R package version 2.5­–6. [https://CRAN.R-project.org/package=vegan](https://cran.r-project.org/package=vegan)

Storchová L, Hořák D (2018) Life‐history characteristics of European birds. Global Ecology and Biogeography 27:400–406.

Wilman H, Belmaker J, Simpson J, la Rosa de C, Rivadeneira MM, Jetz W (2014) EltonTraits 1.0: species-level foraging attributes of the world’s birds and mammals. Ecology 95:2027. https://doi.org/10.1890/13-1917.1
